# Mechanism Analysis of Ethanol Production from Cellulosic Insulating Paper Based on Reaction Molecular Dynamics

**DOI:** 10.3390/polym14224918

**Published:** 2022-11-14

**Authors:** Yufan Fan, Yi Li, Yiyi Zhang, Keshuo Shi

**Affiliations:** 1 Guangxi Power Transmission and Distribution Network Lightning Protection Engineering Technology Research Center, Guangxi University, Nanning 530004, China; 2 School of Mechanical and Electrical Engineering, Liuzhou Vocational & Technical College, Liuzhou 545000, China

**Keywords:** ethanol, cellobiose, ReaxFF, pyrolysis, local hot spots, insulating paper

## Abstract

The paper/oil system is the main component of transformer insulation. Indicator plays a vital role in assessing the aging condition of local hot spots of transformer insulation paper. The cellulosic insulating paper is mainly composed of cellobiose. This study uses the molecular dynamics method based on reactive force field (ReaxFF) to pyrolyze the insulating paper. Various production paths of ethanol were studied at the atomic level through ReaxFF simulations. A model consisting of 40 cellobioses was established for repeated simulation at 500 K–3000 K. Besides, to explore the relationship between the intermediate products and ethanol, the combination model of intermediate products (levoglucosan, acetaldehyde, 2,2-dihydroxyacetaldehyde) was established for repeated simulation. The simulation results showed that the increase in temperature can accelerate the production of ethanol from insulating paper and its pyrolysis intermediate products, which matched the related experimental results. This study can provide an effective reference for the use of ethanol as an indicator to assess the aging condition of the local hot spots of transformers.

## 1. Introduction

In actual operation, transformer winding has a certain temperature field gradient at different heights, which is affected by the winding structure layout and the convection heating-sinking of insulating oil. When the transformer has a serious failure, its internal temperature could exceed 703 °C [1]. Caused by an uneven thermal field, the temperature was not consistent in the different positions of the insulation paper aging degree. Among aging factors, local overheated phenomena were the most serious problem for the aging of insulating paper. During the aging process of the transformer, a large number of gases and chemicals will be gradually produced [2,3]. Dissolved gas analysis in oil is one of the indirect methods to assess the aging condition of the transformer insulation. The content of CO and CO_2_ is directly related to the degree of polymerization (DP) of the insulating paper, and the ratio of two gases (CO_2_/CO) is an important indicator to reflect the degradation degree of cellulose [4]. However, carbon and oxygen-bearing gases are not only produced by the aging of the insulating paper but also produced by the long-term oxidation of the insulating oil. Moreover, air infiltration will also increase the content of CO_2_ in the transformer oil, which interfered with the aging assessment of the transformer insulation. At present, the furfural (2FAL) indicator is one of the most common indicators to assess the aging condition of transformers [5]. 2FAL can reflect the average aging condition of the transformer insulating paper, but it cannot accurately reflect the aging condition of local hot spots, affected by various operating conditions [6]. The aging condition assessed by 2FAL may fail to capture the potential defect and risk of some transformers. Thus, it is necessary to explore a new indicator to assess the aging of the local hot spots of transformers.

Low molecular alcohol, such as ethanol, will be produced during the aging degradation of insulation paper. Ethanol can cross the oil/paper boundary and diffuse into the insulating oil, so it can be theoretically used as an indicator to reflect the aging condition of insulation paper. The higher the content of ethanol in oil, the more serious the aging condition of insulating paper. Using gas chromatography-mass spectrometry (GC-MS), Jalbert et al. identified more than 30 products of the aging degradation of insulating paper [7]. Ethanol is a relatively stable aging degradation product of insulating paper and can be used as an indicator to assess the degradation of insulation performance of insulating paper [7,8]. The physical and chemical properties of ethanol are relatively stable, and the production of ethanol is closely related to the aging and degradation degree of insulating paper. Experimental results show that ethanol could sensitively assess the aging condition of transformer local hot spots of the transformer. However, the micro-mechanism of ethanol production has not been well studied.

In recent years, with the development of molecular simulation technology, chemical microscopic phenomena can be better described. Liu et al. [9] simulated the dynamic process of polymerization-induced self-assembly (PISA) by the dissipative particle dynamics method coupled with the stochastic reaction model. Nies et al. [10] presented a multiscale simulation framework established by combining the coarse-grained molecular dynamics and the reactive Monte Carlo method to study the hyperbranched polymerization of AB_2_ type monomer 4,4-bis-(4′-hydroxyphenyl)pentanoic acid. A molecular reactive dynamics field named ReaxFF was proposed by Van et al. [11]. Compared to the quantum mechanics method and the conventional molecular dynamics method [12,13], the ReaxFF molecular dynamics (MD) can better describe the atomic interactions and dynamic bond scission/formation. The structural changes of reactants and the reaction mechanism can be revealed by using the ReaxFF-MD. With this advantage, the ReaxFF-MD has been increasingly applied in many monomolecular systems and complex molecular systems. By using this method, the initial reaction mechanism of cellulose pyrolysis [14], the high-temperature decomposition of the cellulose molecule [15], the pyrolysis mechanism of Oleic-type Triglycerides [16], and the decomposition mechanism of C_6_F_12_O-CO_2_ gas mixture [17] were studied. The ReaxFF-MD method has extensive application and provides a new direction to study the transformer aging mechanism in the electrical field.

In this paper, the computational chemistry method was used to simulate the insulation paper in the transformer insulation system, and the production of ethanol was tested by experiments during the aging of the insulating paper. The formation path and performance of ethanol were studied, and after that, the feasibility and theoretical basis of ethanol as a new indicator of transformer aging was analyzed.

## 2. Methods

### 2.1. Experiment

Accelerated thermal aging experiments were carried out on the transformer insulating paper to support the subsequent simulation. Firstly, the cellulosic insulating paper was placed in a vacuum for drying pretreatment. Then, the fully dried cellulosic insulating paper was placed in the vacuum immersion tank filled with transformer oil for 24 h. Meet the actual condition of the transformer insulating paper as much as possible. After the oil-immersion steps were completed, the treated cellulosic insulating paper was placed in an aging box for accelerated thermal aging at 50–140 °C for 72 h.

After the accelerated aging experiment was completed, the insulating paper was sampled. To prevent the complex matrix from entering the chromatographic system and reduce the matrix interference, the headspace sample gas chromatography-mass spectrometer (HS-GC-MS) was used to identify the ethanol component in the sample.

### 2.2. Density Functional Theory

Density functional theory (DFT) is a quantum mechanical method to study the electronic structure of multi-electron systems, especially the properties of molecules [18,19]. Because the chemical bond strength of cellobiose is determined by the molecular structure, the DFT method can analyze the characteristics of cellobiose from multiple perspectives, including bonding properties, electrostatic potential, stability, and chemical reactivity. Thus, the stability and reaction site of cellobiose pyrolysis can be analyzed by the electrostatic potential of the molecule, the bond length, and the Mayer bond order, which supports the subsequent ReaxFF-MD study.

**Figure 1:**
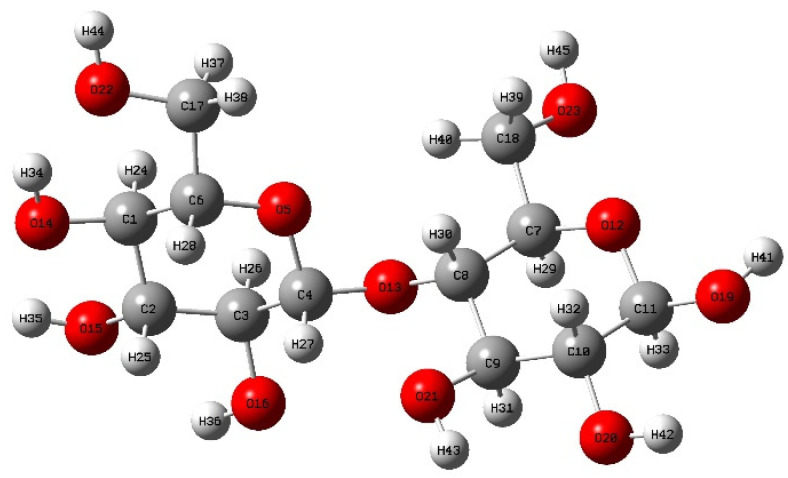
Molecular structure of cellobiose.

Cellobiose molecule is optimized and calculated by the three-parameter hybrid function of Becke based on the correlation function of Lee, Yang, and Parr (B3LYP) with 6-31G (d) basis set using Gaussian 09W packages. The molecular structure of cellobiose is shown in Figure 1. Multiwfn 3.7 [20,21] and VMD 1.9.3 software [22] were used to further analyze the calculated results.

### 2.3. ReaxFF-MD Pyrolysis

The ReaxFF force field is the latest generation of molecular force fields, which has proven to be very suitable for carbohydrate simulation. The ReaxFF force field takes advantage of conventional force fields and chemical reactions. In the ReaxFF force field function, the general system energy *E_system_* is divided into various partial energy terms [11], which is expressed as follows:

(1) $$E_{system}=E_{bond}+E_{lp}+E_{over}+E_{under}+E_{val}+E_{pen}+E_{coa}+E_{C2}+E_{triple}+E_{tors}+E_{conj}+E_{H-bond}+E_{vdWaals}+E_{Coulomb}$$

where *E_bond_* and *E_lp_* denote the bond energy and lone pair electrons terms, respectively. *E_val_*, *E_pen_*, and *E_coa_* denote valence angle and penalty energy terms. *E_tors_* and *E_conj_* denote torsion angle energy and the contribution of conjugation effects to molecular energy, respectively. *E_over_* and *E_under_* denote the atom over-coordination term and under-coordination term, respectively. *E_Hbond_* and *E*_*C*2_ denote the hydrogen bonding term and C2 correction term, respectively. *E_triple_* and *E_vdWaals_* denote triple bonding correction term and nonbonded van der Waals interactions, respectively, and *E_Coulomb_* denotes Coulomb interactions. When these atomic parameters change, the ReaxFF potential function will calculate the distance and the bond level between atoms at the next moment to simulate the process of the chemical reaction.

**Figure 2:**
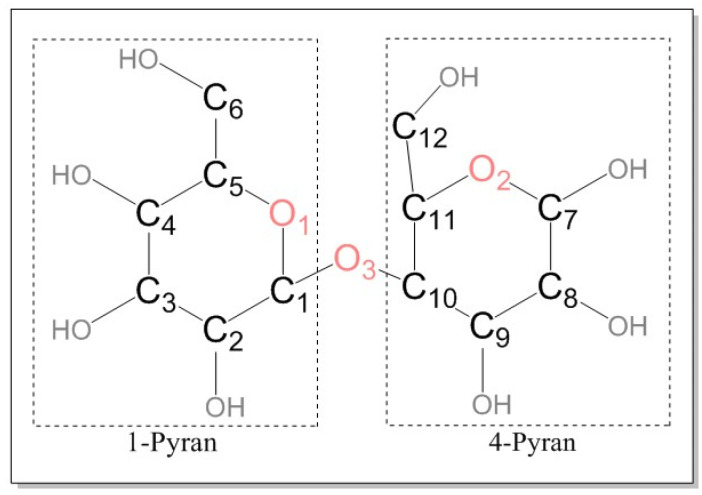
Major labeled atoms of cellobiose.

**Figure 3:**
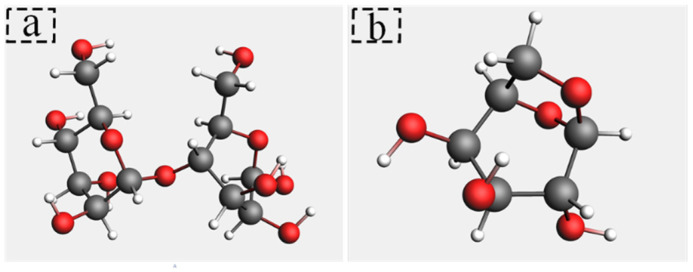
Molecular model. (**a**) is cellobiose; (**b**) is LGA.

**Figure 4:**
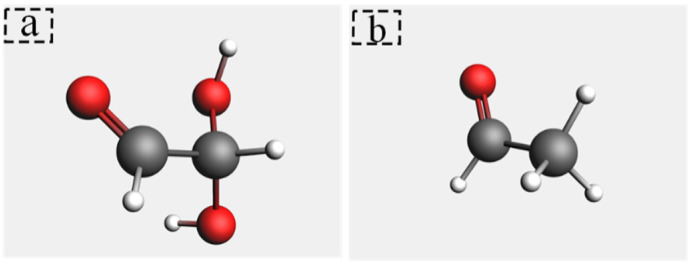
Molecular model. (**a**) is acetaldehyde; (**b**) is 2,2-dihydroxyacetaldehyde.

Cellobiose is the repeating unit of cellulose [23,24]. The DP of new insulation paper is between 1000 and 1200 [25]. It is impractical to establish a molecular model with thousands of DP values. Mezeau simulated the degradation of cellobiose chains with different DP 10, 20, and 40, respectively, and the results appear to be independent of both initial chain length and initial density [26]. To simplify the calculation, a molecular model containing cellobiose with DP = 2 was established. The major atoms of cellobiose are labeled for analysis in Figure 2. The pyrolysis intermediates of cellulose were studied. Research [27] indicated that cellulose would generate a large number of levoglucosan intermediates (LGA). In this study, a molecular model of LGA is established and shown in Figure 3. A large number of vinyl alcohols and 2,2-dihydroxyacetaldehyde were found in the preliminary pyrolysis products of cellobiose. Since vinyl alcohol and acetaldehyde are tautomeric isomers [28], vinyl alcohol will automatically become a mixture of vinyl alcohol and acetaldehyde. Vinyl alcohol is extremely unstable. When vinyl alcohol is produced in the reaction, it would immediately undergo a rearrangement reaction converting C=C into C=O to form a low-energy and stable acetaldehyde. Thus, this paper discusses acetaldehyde instead of vinyl alcohol, and molecular models of acetaldehyde and 2,2-dihydroxyacetaldehyde are established (Figure 4).

**Figure 5:**
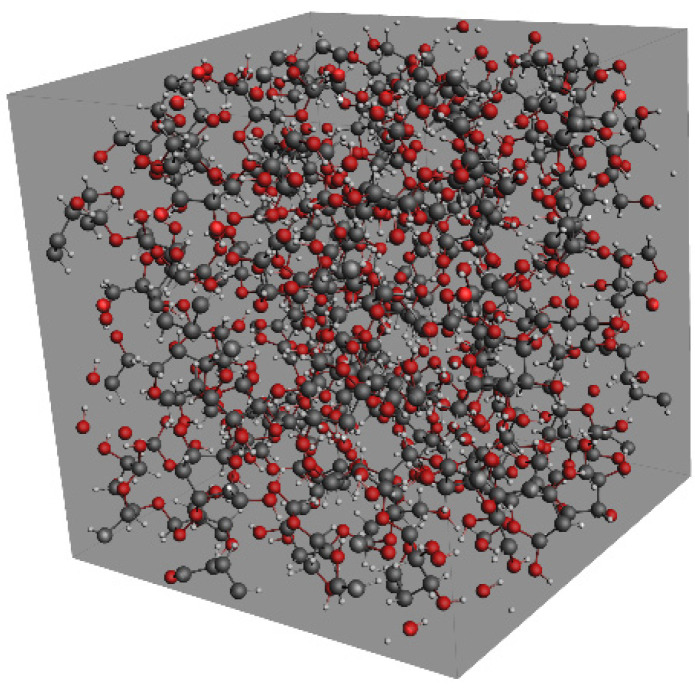
Molecular model of 40 cellobioses.

In this paper, the ReaxFF module of AMS software was used to simulate the pyrolysis of insulating paper in the transformer [29,30]. After the geometry optimization, an initial amorphous cell (2.42 nm × 2.48 nm × 2.39 nm) containing 40 cellobioses was established. Its energy was minimized and the density of the model was adjusted to 1.59 g/cm^3^ [31]. After the above treatments, the established molecular model shown in Figure 5 was similar to the actual one. Besides, an initial amorphous cell containing 150 LGA and an initial amorphous cell containing 100 acetaldehydes and 100 2,2-dihydroxyacetaldehydes were built, which aimed to explore the relationship between the intermediate products and ethanol. Under the canonical ensemble (NVT), these models were relaxed to reach the equilibrium state gradually.

This paper only contained C, H, and O elements, so the CHO field of the ReaxFF module was used in the simulation [30], and the canonical ensemble (NVT) was used to simulate the pyrolysis. Based on the transition-state theory, the temperature-accelerated dynamics method proposed in research [32], can speed up the molecular transition by raising the temperature. However, this method only allows occuring the events of original temperature. Compared with the conventional molecular dynamics, the simulation time scale of the temperature accelerated dynamics method can be extended greatly on the order of magnitude. It also retains the correct dynamic behavior of the original temperature. To simplify the calculation, the cellobiose molecular model was simulated at the temperature of 500–3000 K. The LGA model and the combined molecular model of acetaldehyde and 2,2-dihydroxyacetaldehyde were simulated at temperatures of 2600 K, 2800 K and 3000 K, respectively. The pyrolysis time was set as 100 ps, and the time for each step was set as 0.1 fs. Then, the force bias Monte-Carlo (fbMC) was mixed in to increase the randomness of the experiment, which is closer to the actual situation [33,34,35,36]. Data were recorded every 10 fs. The other parameters except temperature should be consistent to ensure the pyrolysis reliability of each model.

## 3. Results and Discussion

### 3.1. Experimental Results

**Figure 6:**
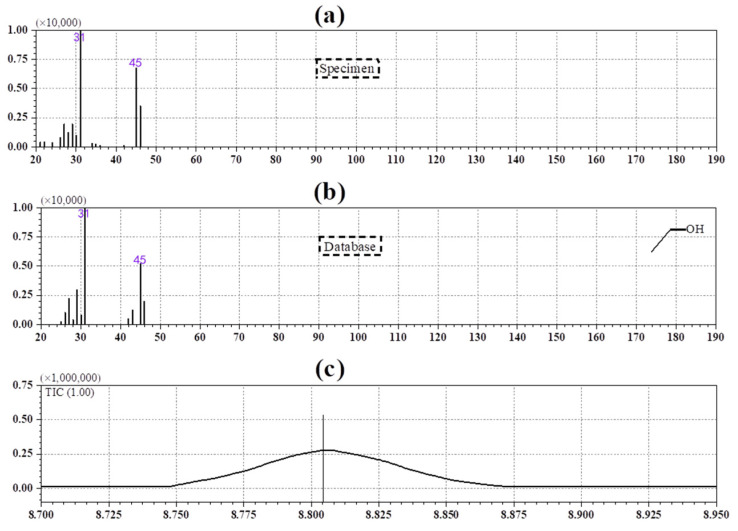
HS-GC-MS analysis of ethanol in the oil-impregnated insulating paper: (**a**) a sample of aged fragments; (**b**) the NIST database; (**c**) the production time of ethanol.

The HS-GC-MS was used to sample ethanol from cellulose pyrolysis. When the temperature is over 100 °C, large contents of ethanol are produced from the aged insulating paper. Figure 6a shows a sample of aged fragments of oil-impregnated insulating paper. Figure 6b shows the pyrolysis product (i.e., ethanol) matched in the NIST database. Figure 6c shows the production time of ethanol in the total ion flow diagram. It can be seen that ethanol is generated and existed stably in the pyrolysis, so ethanol can be feasibly used as a new indicator of insulating paper aging. This result is consistent with the previous experimental results. To clearly describe the formation mechanism of ethanol during insulating paper aging, the formation of ethanol was studied by the following simulation.

### 3.2. Molecular Structure of Cellobiose

#### 3.2.1. Bond Length and Mayer Bond Order Analysis

The bond length of the molecular chemical bond is not only the basic parameter of molecular structure, but also the basic parameter of the strength and properties of the molecular chemical bond. For the chemical bond between two atoms, bond interaction is strong when the value of the bond length is small. Mayer bond order is the parameter used in molecular orbital methods to indicate the bonding strength of two adjacent atoms [37]. The larger the bond order, the more stable the bond. For the same kind of bond, the bond length is negatively correlated with the bond order—since the longer the bond length, the smaller the bond order. The bond length and Mayer bond order of cellobiose were analyzed by Multiwfn and listed in Table 1. In the cellobiose molecule (Figure 1), the Mayer bond order of C_4_-O_5_, C_7_-O_12_ and C_8_-O_13_ was three of the lowest among all chemical bonds. These bonds in the cellobiose molecule are most easily cleaved under local hot spots. On the contrary, the Mayer bond order of C_6_-C_17_ and C_7_-C_18_ indicated that these bonds are the most stable.

#### 3.2.2. Electrostatic Potential on the Molecular Surface

Electrostatic potential plays a unique role in intermolecular interactions and intermolecular reaction sites. Electrostatic potential consists of positive contributions and negative contributions. The positive contribution is generated by the positively charged nuclear, and the negative contribution is generated by the negatively charged electron. In this paper, the electrostatic potential was used to predict reaction sites and analyze intermolecular interactions. Calculations were completed by Gaussian, and results were analyzed and drawn by Multiwfn and VMD, respectively.

**Figure 7:**
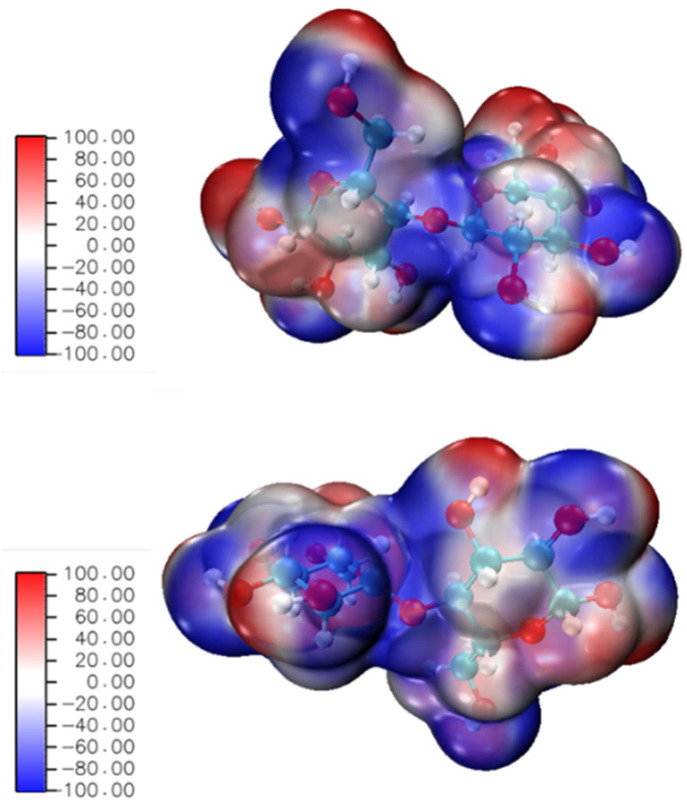
Electrostatic potential of cellobiose molecule.

At the beginning of chemical reactions, through electrostatic attraction, the process of molecules approaching each other is closely related to the electrostatic effect of molecules on their outer surface (van der Waals surface and beyond). Thus, the location of chemical reactions can be predicted by analyzing the distribution of electrostatic potential on the van der Waals surface. If the negative (or positive) value of the electrostatic potential was larger, more electrophilic (or nucleophilic) reactions would occur in the corresponding atoms. In Figure 7, configurations of cellobiose molecule are the top view (above) and side view (below), and the red part denotes where the electrostatic potential is positive, otherwise, the blue part denotes where the electrostatic potential is negative. The C-O bond and glycoside bond were in the blue part, which was prone to electrophilic reactions. H atom was in the red part, which was prone to nucleophilic reactions. The above results showed that the C-O bond and H atom in the cellobiose molecule were weak and had robust reactivity. The pyrolysis of the cellobiose molecule may first occur on the C-O bond and H atom.

### 3.3. ReaxFF-MD of Cellobioses

#### 3.3.1. Production of Ethanol in the Pyrolysis

**Figure 8:**
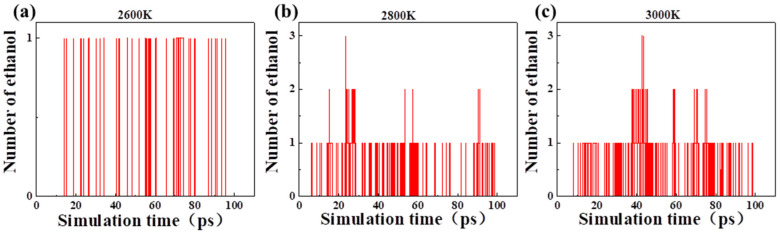
Production of ethanol molecules in the pyrolysis of cellobiose at different temperatures: (**a**) 2600 K, (**b**) 2800 K and (**c**) 3000 K.

In the pyrolysis of cellobiose, the production of ethanol molecules changed with time at different temperatures (Figure 8). No ethanol was found when the temperature was lower than 2400 K, and some ethanol molecules appeared at 2600 K, 2800 K, and 3000 K. When the temperature was higher than 2600 K, ethanol was stably generated. With the increase of temperature, the production rate and yield of ethanol increased obviously. It is revealed that the production of ethanol was closely related to temperature. If ethanol could be used as a new indicator for aging assessment during the actual operation of the oil-immersed transformer, the aging status of local hot spots of winding could be more accurately characterized, especially when the local high-temperature thermal fault occurs.

**Figure 9:**
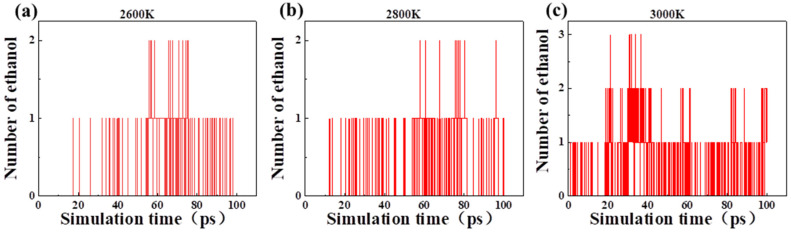
Production of ethanol molecule in the pyrolysis of LGA at different temperatures: (**a**) 2600 K; (**b**) 2800 K and (**c**) 3000 K.

As an important intermediate, LGA also produced ethanol in pyrolysis, and the production of ethanol molecules changed with time at different temperatures (Figure 9). With the increase in pyrolysis temperature, the production rate of ethanol was accelerated. However, there was no significant difference in the content of the production. It is indicated that the increase in temperature will only accelerate the formation of ethanol but not increase the content of ethanol. LGA was similar to cellobiose. With the increase in temperature, the formation of ethanol was accelerated, which proved that ethanol indicator could reflect local hot spot faults.

**Figure 10:**
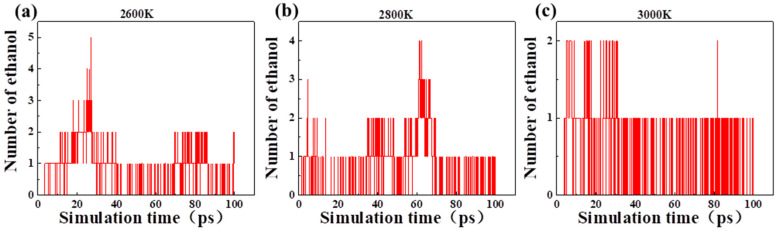
Production of ethanol molecule in the pyrolysis of acetaldehyde and 2,2-dihydroxyacetaldehyde: (**a**) 2600 K; (**b**) 2800 K; (**c**) 3000 K.

Figure 10 shows the production of ethanol in the pyrolysis of acetaldehyde and 2,2-dihydroxyacetaldehyde. Acetaldehyde and 2,2-dihydroxyacetaldehyde are pyrolysis intermediates with a large number, and ethanol is produced by their interaction. However, the increase in temperature did not accelerate the formation of ethanol, which was completely different from the pyrolysis of cellobiose and LGA. Ethanol existed throughout the pyrolysis process proving that ethanol indicator can be correlated with the aging state.

#### 3.3.2. Analysis of Ethanol Formation Path

**Figure 11:**
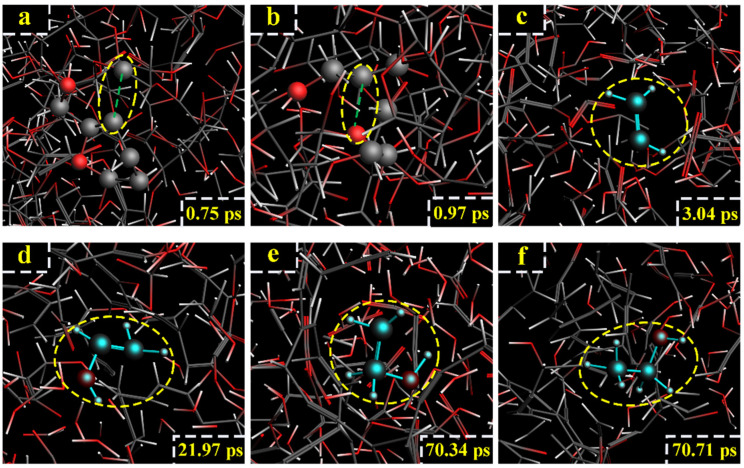
Path I for the production of ethanol. (**a**–**f**) are the procedure of cellobiose to produce ethanol at 2600 K.

**Figure 12:**
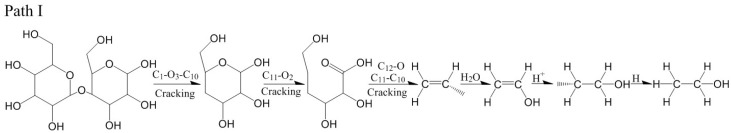
Relevant generation path I of ethanol at 2600 K.

Through the process visualization module of simulation software, the fracture and formation of reactant molecular bonds were observed, and the formation path of ethanol molecules at different simulation moments was analyzed. To facilitate the analysis, the simulated image is partially enlarged. As shown in Figure 2, the specific molecular group is highlighted or marked with a dotted circle, and the broken chemical bond is marked with a dotted line. This section analyzes the labeled atoms shown in Figure 2. The generation path of ethanol is as follows:

For the model of cellobiose at 2600 K, by tracing the source of the formation of ethanol molecules, it can be found that ethanol was mainly formed through C_11_ and C_12_. At 0.75 ps, the glucosidic bond (C_1_-O_3_-C_10_) broke (Figure 11a). At 0.97 ps, the 4-Pyran ring broke after the C_11_-O_2_ bond broke (Figure 11b). At 3.04 ps, the C_12_-O and C_11_-C_12_ bond broke, and -CH=CH_2_ was generated (Figure 11c). At 21.97 ps, -CH=CH_2_ reacted with H_2_O and H^+^, vinyl alcohol (CH_2_=CHOH) was generated (Figure 11d). At 70.34 ps, vinyl alcohol reacted with H^+^ to generate -CH_2_CH_2_OH (Figure 11e). Finally, at 70.71 ps, -CH_2_CH_2_OH captured H atom on the nearby hydroxyl group, and ethanol was generated (Figure 11f). The relevant paths are shown in Figure 12.

**Figure 13:**
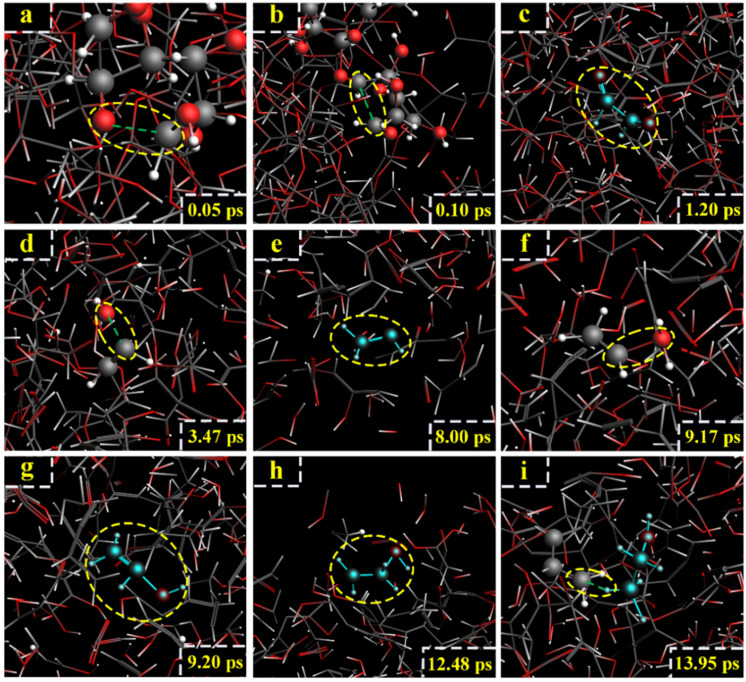
Path II for the production of ethanol. (**a**–**i**) are the procedure of cellobiose to produce ethanol at 2800 K.

**Figure 14:**
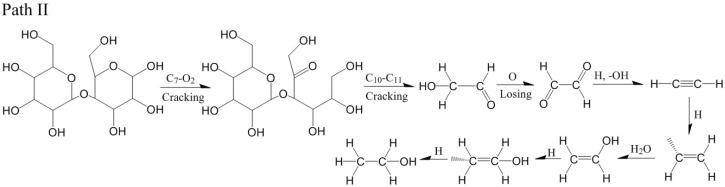
Relevant generation path II of ethanol at 2800 K.

For the model of cellobiose at 2800 K, the formation of ethanol was also related to C_11_ and C_12_. However, the generation path of ethanol at 2800 K was different from that at 2600 K. At 0.05 ps, the C_7_-O_2_ broke (Figure 13a). At 0.1 ps, the C_11_-C_12_ bond broke, and glycolic aldehyde (CH_2_OH-CHO) was generated (Figure 13b). At 1.2 ps, glycolic aldehyde lost an H atom, and glyoxal (CHO-CHO) was generated (Figure 13c). At 3.47 ps, glyoxal reacted with H^+^, -OH was lost, and acetylene was generated (Figure 13d). At 8 ps, because of the active chemical properties of acetylene, acetylene captured an H atom to generate -CH-CH_2_ (Figure 13e). At 9.17 ps, -CH-CH_2_ captured the -OH of H_2_O due to instability (Figure 13f). At 9.2 ps, -CH-CH_2_ captured the -OH to generate vinyl alcohol (Figure 13g). At 12.48 ps, vinyl alcohol captured H atom, and -CH_2_-CH_2_OH was generated (Figure 13h). At 13.95 ps, -CH_2_-CH_2_OH captured H atom, and ethanol was finally generated (Figure 13i). The relevant paths (Figure 14).

**Figure 15:**
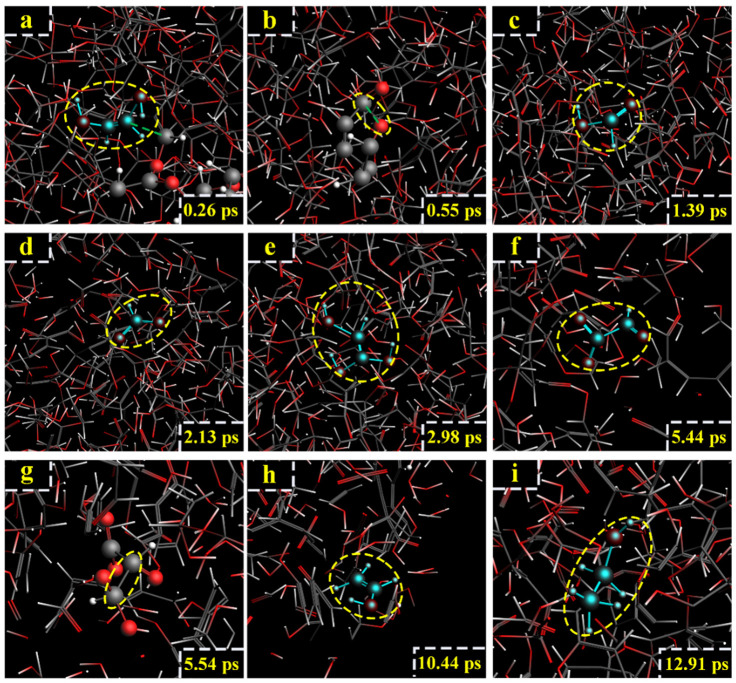
Path III for the production of ethanol. (**a**–**i**) are the procedure of cellobiose to produce ethanol at 3000 K.

**Figure 16:**
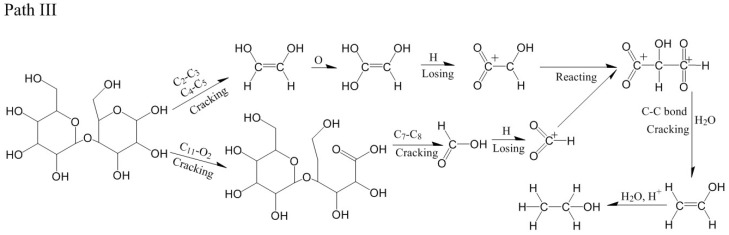
Relevant path III of ethanol generation at 3000 K.

For the model of cellobiose at 3000 K, the main part of ethanol formation came from C_4_ and C_7_. The path was different from the path I and path II. At 0.26 ps, the C_2_-C_3_ and C_4_-C_5_ bond broke, and the CHOH=CHOH of C_4_ atom was generated (Figure 15a). At 0.55 ps, the 4-Pyran ring broke after the C_11_-O_2_ bond broke (Figure 15b). At 1.39 ps, the C_7_-C_8_ bond broke, and formic acid (HCOOH) of C_7_ atom was generated (Figure 15c). At 2.13 ps, formic acid lost an H atom, and -CHO_2_ was generated (Figure 15d). At 2.98 ps, CHOH=CHOH captured an O atom to generate C(OH)_2_=CHOH (Figure 15e). At 5.44 ps, C(OH)_2_=CHOH lost two H atoms, and -C=(O)_2_-CHOH was generated (Figure 15f). At 5.54 ps, -CHO_2_ reacted with -C=(O)_2_-CHOH, and the C-C bond was formed to generate -C=(O)_2_-CHOH-CH^+^=(O)_2_ (Figure 15g). At 10.44 ps, the -C=(O)_2_ fell off -C=(O)_2_-CHOH-CH^+^=(O)_2_ and reacted with H_2_O, and vinyl alcohol was generated (Figure 15h). At 12.91 ps, vinyl alcohol reacted with H^+^ and H_2_O, and ethanol was finally generated (Figure 15i). The relevant paths are shown in Figure 16.

**Figure 17:**
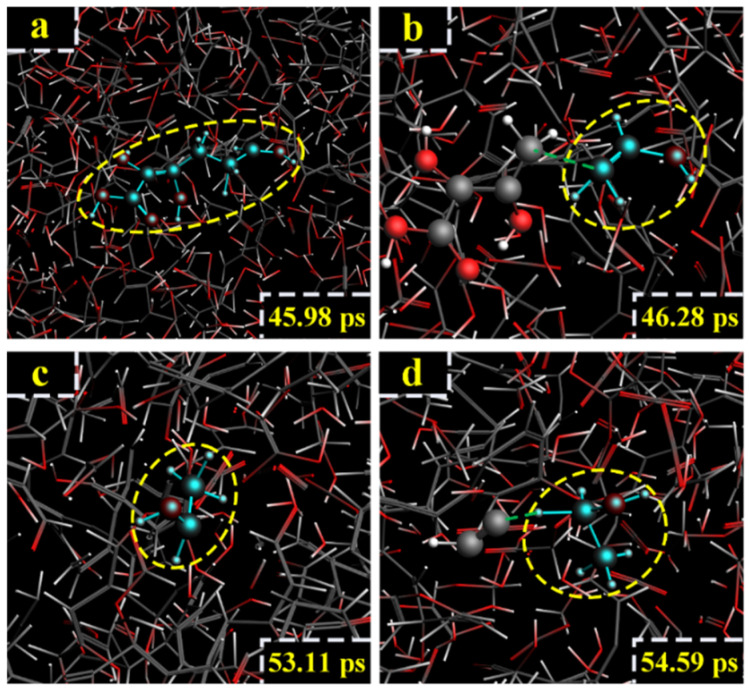
Path IV for the production of ethanol. (**a**–**d**) are the procedure of LGA to produce ethanol at 2600 K.

**Figure 18:**
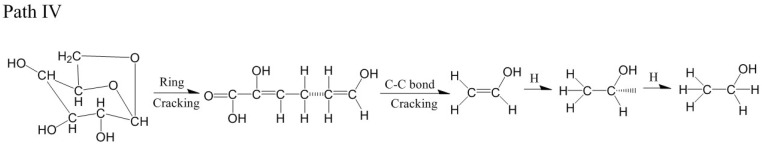
Relevant path IV of ethanol generation at 2600 K.

In the LGA model at 2600 K, vinyl alcohol was a vital intermediate. At 45.98 ps, the ring of LGA broke, and the chain compound was generated (Figure 17a). At 46.28 ps, vinyl alcohol was generated after the C-C bond of the chain compound broke (Figure 17b). At 53.11 ps, vinyl alcohol captured an H atom to generate -CHOH-CH_3_ (Figure 17c). At 54.59 ps, -CHOH-CH_3_ captured an H atom, and ethanol was finally generated (Figure 17d). The relevant paths are shown in Figure 18.

**Figure 19:**
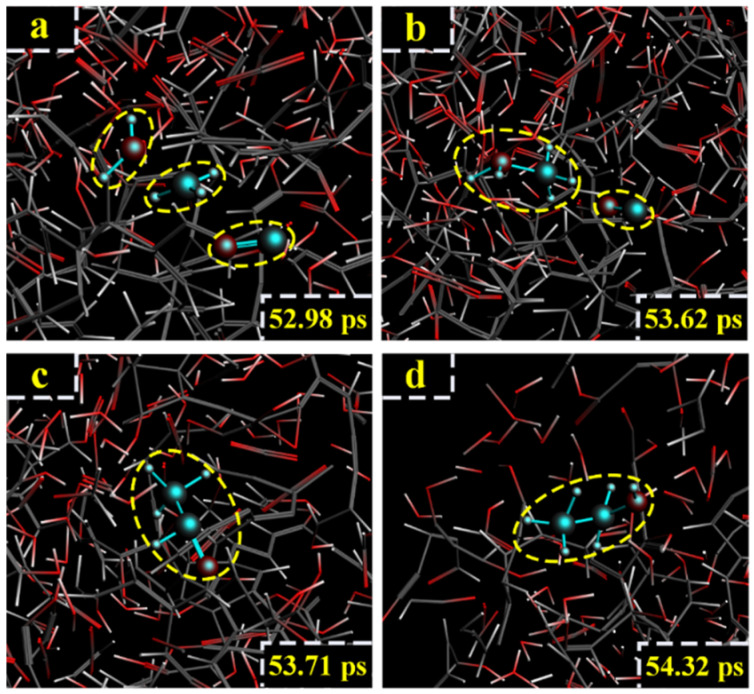
Path V for the production of ethanol. (**a**–**d**) are the procedure of LGA to produce ethanol at 2800 K.

**Figure 20:**
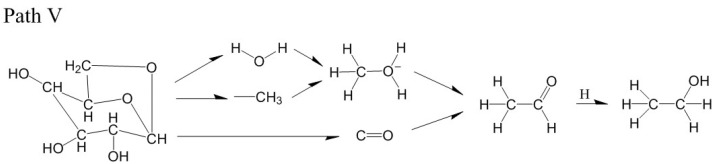
Relevant path V of ethanol generation at 2800 K.

For the LGA model at 2800 K, the formation path of ethanol was different from path IV. At 52.98 ps, H_2_O, -CH_3_ and CO were generated after the ring of LGA broke (Figure 19a). At 53.62 ps, H_2_O reacted with -CH_3_, and -O(H)_2_-CH_3_ was generated (Figure 19b). At 53.71 ps, -O(H)_2_-CH_3_ reacted with CO, and aldehyde was generated (Figure 19c). At 54.32 ps, aldehyde captured an H atom, and ethanol was finally generated (Figure 19d). The relevant paths are shown in Figure 20.

**Figure 21:**
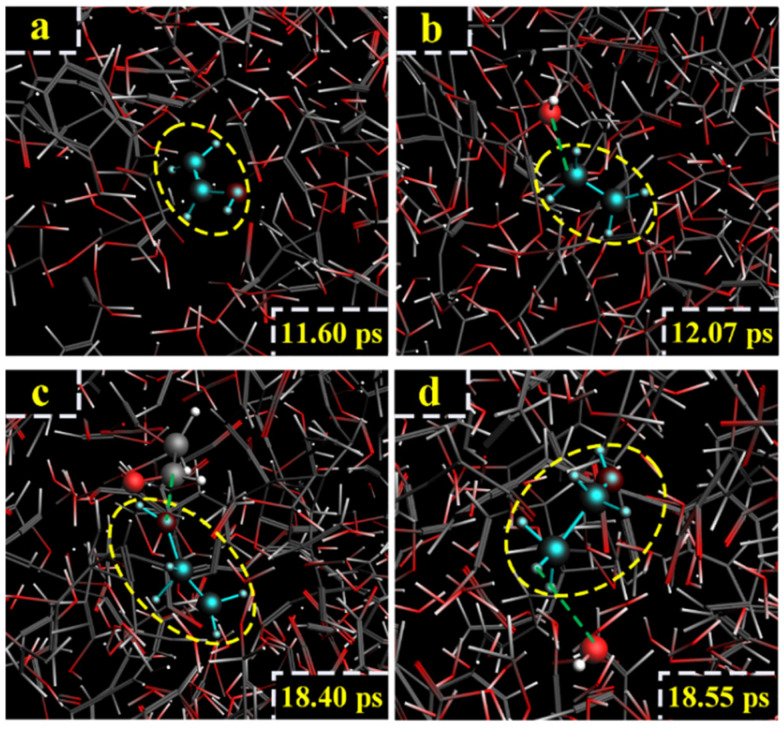
Path VI for the production of ethanol. (**a**–**d**) are the procedure of LGA to produce ethanol at 3000 K.

**Figure 22:**
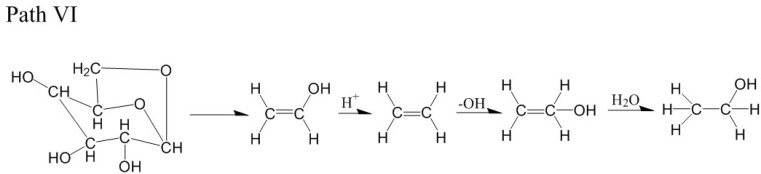
Relevant path VI of ethanol generation at 3000 K.

For the LGA model at 3000 K, the formation path of ethanol was similar to path IV, with the generation of vinyl alcohol. At 11.6 ps, LGA generated vinyl alcohol, which is shown in Figure 21a. At 12.07 ps, vinyl alcohol reacted with H^+^, and ethylene was generated (Figure 21b). At 18.40 ps, ethylene captured -OH, and CH_2_=CH_2_OH^−^ was generated (Figure 21c). At 18.55 ps, CH_2_=CH_2_OH^−^ reacted with H_2_O, and ethanol was finally generated (Figure 21d). The relevant paths are shown in Figure 22.

**Figure 23:**
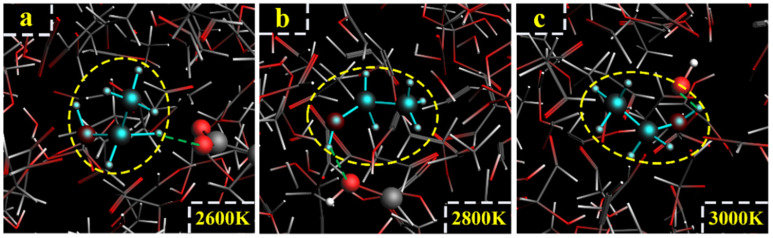
Path VII for the production of ethanol. (**a**–**c**) are the procedure of acetaldehyde and 2,2-dihydroxyacetaldehyde to produce ethanol at 3000 K.

**Figure 24:**
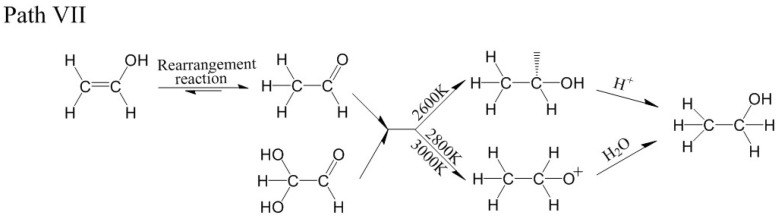
Relevant path VII of ethanol generation.

For the model of acetaldehyde and 2,2-dihydroxyacetaldehyde at 2600 K, 2800 K and 3000 K, there were two main formation paths of ethanol. For one path, acetaldehyde reacted with 2,2-dihydroxyacetaldehyde to generate -CHOH-CH_3_, and -CHOH-CH_3_ captured H^+^ to generate ethanol (Figure 23a). For another path, acetaldehyde reacted with 2,2-dihydroxy-acetaldehyde to generate CH_3_-CH_2_O^+^, and ethanol was generated in the reaction of CH_3_-CH_2_O^+^ and H_2_O (Figure 23b,c). The relevant paths are shown in Figure 24.

**Figure 25:**
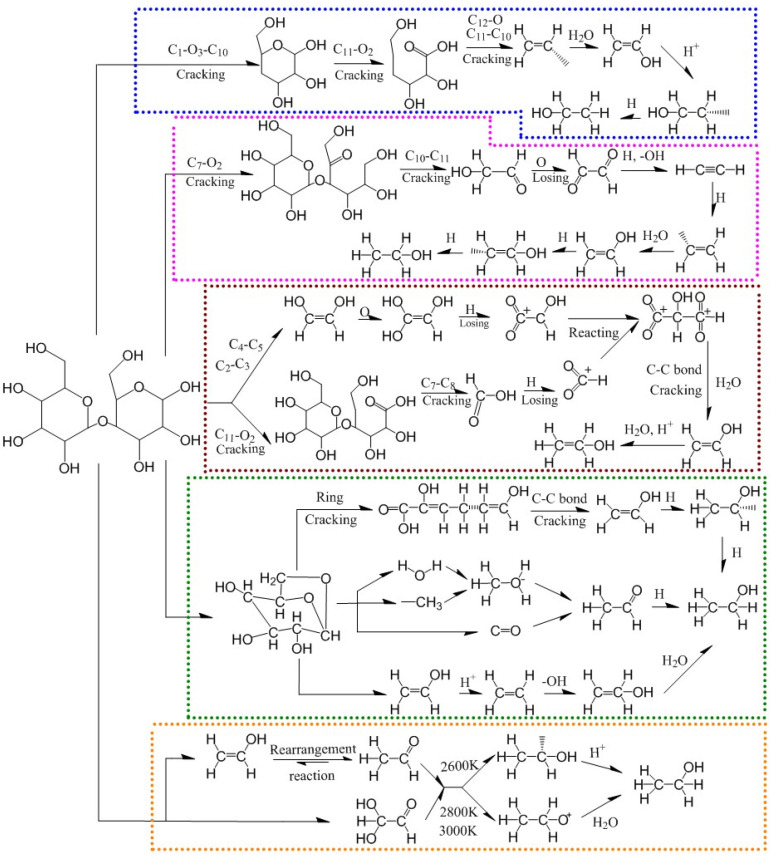
The pyrolysis path of cellobiose to generate ethanol.

By analyzing the pyrolysis path, the pyrolysis mechanism of cellobiose and its intermediate products were summarized and shown in Figure 25.

### 3.4. Kinetic Analysis of Cellobiose Pyrolysis

In this study, the first-order pyrolysis kinetics was used to verify the accuracy of the ReaxFF-MD simulated pyrolysis. The first-order pyrolysis kinetics using the consumption rate of reactants has been widely studied [38,39,40], and these studies assumed that the reactants had been completely pyrolyzed in the kinetic model. The kinetic characteristics of cellobiose pyrolysis at 2600–3000 K were analyzed in this paper. According to the results of first-order pyrolysis kinetics, the reliability of the simulation was examined.

Under the premise of ensuring accuracy, the concentration of the cellobiose reactant was replaced by the molecular number of cellobiose to effectively calculate the rate constant. By using Equation (2), the number of molecules (*N*_t_) and simulation time (*t*) can be linearly fitted. *N*_0_ denotes the original number of cellobiose molecules, and it is equal to 40 in this study. The rate constant *k* under constant temperature *T* was calculated. Besides, the natural logarithm of rate constant *k* (ln*k*) and the reciprocal of constant temperature *T* (1/*T*) was used for linear fitting. The activation energy E_a_ and pre-exponential factor A were calculated through the Arrhenius expression shown in Equation (3). R is the molar gas constant, and its value is approximately equal to 8.3144 J/(mol·K).

(2) $$\ln N_{t}-\ln N_{0}=kt$$

(3) $$\ln k=-\frac{E_{a}}{R}\frac{1}{T}+\mathrm{lnA}$$

**Figure 26:**
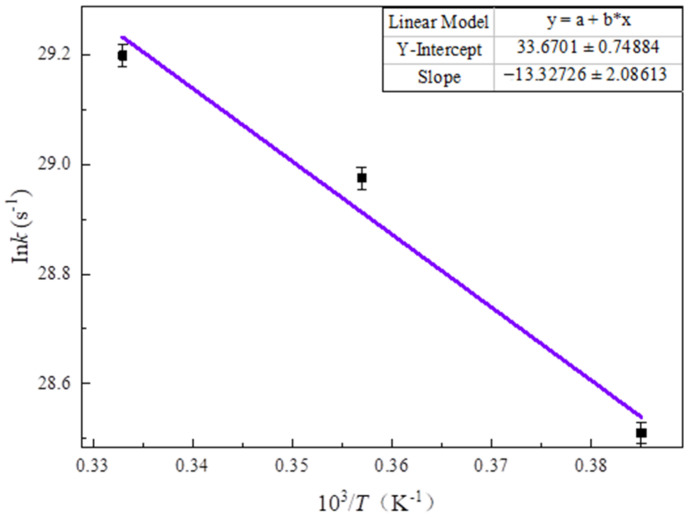
Fitted napierian logarithm of the rate constant *k* versus inverse temperature *T* obtained from simulations of cellobiose pyrolysis at 2600–3000 K.

The relevant data of simulation results were calculated by Equation (2) to calculate the rate constant *k* for many times. Then, In*k* and 1/*T* were fitted linearly by using Equation (3), and the result is shown in Figure 26. The fitting slope was −13.32 × 10^3^ K·s^−1^, and the Y-intercept was 33.67 s^−1^. By calculation, the activation energy E_a_ and pre-exponential factor A were 110.75 kJ·mol^−1^ and 4.19 × 10^14^ s^−1^, respectively. The pyrolysis kinetics of cellulose has been investigated in many studies considering various kinetic models, sample materials and reaction conditions. It was reported that activation energies ranged from 48 kJ·mol^−1^ to 282 kJ·mol^−1^, and the pre-exponential factor ranged from 180 s^−1^ to 1.33 × 10^23^ s^−1^ [15,27]. Comparing the calculated results with the previous experimental results, it can be concluded that this simulation study was reliable, because the pyrolysis results were within the range of experimental results. Meanwhile, the temperature was much higher than that of the experiment, and the time scale was much smaller than that of the experiment, so high temperature did not distort the simulation. It is proved that reaction molecular dynamics is a reliable tool for studying transformer insulation paper aging.

## 4. Conclusions

In this study, the accelerated aging experiment was carried out, and the DFT and the ReaxFF-MD method were used to study the pyrolysis of insulating paper. Firstly, ethanol was detected in pyrolysis products by accelerated aging experiments. It was found that ethanol can exist stably in local hot spots. On this basis, by using the DFT method, the properties of cellobiose were studied, and the reaction position of cellobiose was predicted. Moreover, by using the ReaxFF-MD method, the formation path of ethanol at different temperatures was analyzed based on the formation or fracture of chemical bonds at the atomic level. Finally, the first-order kinetic model was used to verify the accuracy of the ReaxFF-MD pyrolysis. The results were consistent with previous experiments, which indicated that the ReaxFF-MD method could be used to study the aging process of insulating paper.

The bond length and Mayer bond order can reveal the chemical reactivity characteristics of cellobiose, which provides an effective basis for the interpretation of cellobiose pyrolysis. The C-O bond was susceptible to cleavage. The electrostatic potential predicted the C-O bond, and H atom was easy to react to because of its robust reactivity.

The ReaxFF-MD results showed that ethanol mainly came from C_11_ and C_12_, but partly from C_4_ and C_7_. Cellobiose can produce ethanol directly through pyrolysis. The intermediate products (LGA, acetaldehyde and 2,2-dihydroxyacetaldehyde) of cellobiose can also indirectly produce ethanol. According to the formation paths, vinyl alcohol was an important intermediate conversion medium to generate ethanol. The simulation of insulating paper aging was verified by the first-order kinetic model. The calculated activation energy Ea and the pre-exponential factor A are 110.75 kJ·mol^−1^ and 4.19 × 10^14^ s^−1^, respectively, which are in line with the previous experimental results and the Arrhenius’ law. The pyrolysis results show that the ReaxFF-MD method can describe the multi-channel pyrolysis path and reaction trend of cellulosic insulating paper in detail. The kinetic analysis indicates that the ReaxFF-MD method can be feasibly used to study rapid pyrolysis and reveal the complex pyrolysis mechanism of insulating paper at the atomic level.

According to the analysis of pyrolysis at different temperatures, ethanol was mainly generated above 2600 K. In addition, the formation of ethanol can be accelerated by increasing the temperature. When the transformer has a local hot spot fault, its temperature is generally higher than the temperature near the hot spot. Local hot spots produced more ethanol than other locations. Therefore, ethanol can be used as an assessment indicator of the aging condition of transformers. Ethanol can reflect the local hot spots of transformer insulation paper more accurately, especially when the local high-temperature thermal fault occurs. However, compared with furfural, it has poor performance in general thermal aging. Therefore, it is necessary to combine ethanol with other indicators for further research to make up for the deficiency of the performance of ethanol. This work provides the theoretical basis for ethanol as a new indicator to assess the aging condition of the local hot spots of transformers.

## Figures and Tables

**Table 1:** Bond length and Mayer bond order of cellobiose.

Bond	Bond Length	Mayer Bond Order	Bond	Bond Length	Mayer Bond Order
C_1_-C_2_	1.5106	0.8705	C_4_-O_13_	1.4047	0.8371
C_2_-C_3_	1.5137	0.8626	C_8_-O_13_	1.4401	0.7902
C_3_-C_4_	1.5137	0.8601	C_1_-O_14_	1.4322	0.8681
C_4_-O_5_	1.4443	0.7749	C_2_-O_15_	1.4358	0.8602
O_5_-C_6_	1.4386	0.8065	C_3_-O_16_	1.4305	0.8653
C_6_-C_1_	1.5254	0.8197	C_6_-C_17_	1.5182	0.9207
C_7_-C_8_	1.5288	0.8469	C_17_-C_22_	1.4497	0.8175
C_8_-C_9_	1.5172	0.8785	C_9_-O_21_	1.4383	0.8574
C_9_-C_10_	1.5135	0.8603	C_10_-O_20_	1.4340	0.8489
C_10_-C_11_	1.5069	0.8285	C_11_-O_19_	1.4101	0.8729
C_11_-O_12_	1.4224	0.8229	C_7_-C_18_	1.5105	0.9197
C_7_-O_12_	1.4473	0.7936	C_18_-O_23_	1.4406	0.8663

## Data Availability

The data presented in this study are available on request from the corresponding author.
